# Chikungunya Virus Transmission at Low Temperature by *Aedes albopictus* Mosquitoes

**DOI:** 10.3390/pathogens8030149

**Published:** 2019-09-12

**Authors:** B. M. C. Randika Wimalasiri-Yapa, Liesel Stassen, Wenbiao Hu, Laith Yakob, Elizabeth A. McGraw, Alyssa T. Pyke, Cassie C. Jansen, Gregor J. Devine, Francesca D. Frentiu

**Affiliations:** 1 Institute of Health and Biomedical Innovation, School of Biomedical Sciences, Queensland University of Technology, Brisbane, QLD 4001, Australia; 2 Department of Medical Laboratory Sciences, Faculty of Health Sciences, The Open University of Sri Lanka, Colombo 10250, Sri Lanka; 3 Institute of Health and Biomedical Innovation, School of Public Health and Social Work, Queensland University of Technology, Brisbane, QLD 4059, Australia; 4 Department of Disease Control, Faculty of Infectious & Tropical Diseases, The London School of Hygiene & Tropical Medicine, London WC1H 9SH, UK; 5 Department of Entomology, Center for Infectious Disease Dynamics, Huck Institutes of the Life Sciences, Pennsylvania State University, State College, PA 16802, USA; 6 Public Health Virology Laboratory, Forensic and Scientific Services, Queensland Health, Coopers Plains, QLD 4108, Australia; 7 Communicable Diseases Branch, Queensland Health, Herston, QLD 4006, Australia; 8 Mosquito Control Laboratory, QIMR Berghofer Medical Research Institute, Brisbane, QLD 4006, Australia

**Keywords:** chikungunya, *Aedes albopictus*, vector competence, temperature, extrinsic incubation period

## Abstract

*Aedes albopictus* is an important vector of chikungunya virus (CHIKV). In Australia, *Ae. albopictus* is currently only known to be present on the islands of the Torres Strait but, should it invade the mainland, it is projected to spread to temperate regions. The ability of Australian *Ae. albopictus* to transmit CHIKV at the lower temperatures typical of temperate areas has not been assessed. *Ae. albopictus* mosquitoes were orally challenged with a CHIKV strain from either Asian or East/Central/South African (ECSA) genotypes (10^7^ pfu/mL), and maintained at a constant temperature of either 18 °C or 28 °C. At 3- and 7-days post-infection (dpi), CHIKV RNA copies were quantified in mosquito bodies, and wings and legs using real time polymerase chain reaction (qRT-PCR), while the detection of virus in saliva (a proxy for transmission) was performed by amplification in cell culture followed by observation of cytopathic effect in Vero cells. Of the ≥95% of *Ae. albopictus* that survived to 7 dpi, all mosquitoes became infected and showed body dissemination of CHIKV at both temperatures and time points. Both the Asian and ECSA CHIKV genotypes were potentially transmissible by Australian *Ae. albopictus* at 28 °C within 3 days of oral challenge. In contrast, at 18 °C none of the mosquitoes showed evidence of ability to transmit either genotype of CHIKV at 3 dpi. Further, at 18 °C only *Ae. albopictus* infected with the ECSA genotype showed evidence of virus in saliva at 7 dpi. Overall, infection with the ECSA CHIKV genotype produced higher virus loads in mosquitoes compared to infection with the Asian CHIKV genotype. Our results suggest that lower ambient temperatures may impede transmission of some CHIKV strains by *Ae. albopictus* at early time points post infection.

## 1. Introduction

Chikungunya is a vector-borne disease that has caused recent outbreaks in tropical and sub-tropical regions of the world [1]. Though the disease is rarely fatal, it causes incapacitating arthralgia which can persist for months after infection [2]. There are three genotypes of chikungunya virus (CHIKV), classified according to original geographical distributions, known as Asian, East/Central/South African (ECSA) and West African [3]. The primary vector of CHIKV is *Aedes aegypti* [4]. A secondary vector, the Asian tiger mosquito, *Ae. albopictus* has been implicated in large, unprecedented outbreaks in tropical areas, and in the geographical expansion of CHIKV to temperate countries such as France and Italy [5,6]. Indeed, *Ae. albopictus* was the primary vector during the explosive outbreak that occurred on Reunion island [7] in 2005/2006, resulting in hundreds of thousands of cases [8]. The ability of *Ae. albopictus* to transmit the CHIKV strain, belonging to the ECSA genotype, was partly enhanced by mutations in the viral envelope 1 (E1) protein. Further transmission and dissemination of the mutated CHIKV in the Indian Ocean region led to the appearance of a new virus subgroup, the Indian Ocean Lineage (IOL). Mutations appearing in this lineage were later shown to allow replication to higher titers in the vector *Ae. albopictus* [9,10]. 

Australia is a non-endemic country for CHIKV. However, the risk of CHIKV introductions into Australia is high due to the close proximity of CHIKV-endemic neighboring countries in the Asia-Pacific region [11], a dramatic increase in imported CHIKV viremic cases [12] and the presence of established populations of both *Ae. aegypti* and *Ae. albopictus* mosquitoes in parts of the state of Queensland. There is also the potential for *Ae. albopictus* mosquitoes, currently confined to the Torres Strait in north Queensland, to extend their geographical range and become established on the mainland [13,14]. Australian *Ae. albopictus* mosquitoes have also been shown to be highly competent vectors of the ECSA CHIKV genotype in the laboratory [15]. The Asian CHIKV genotype actively circulates in Australia’s neighboring region [11] and recently caused an outbreak in New Caledonia [16]. A recent review on the vector competence of *Aedes* spp. for CHIKV indicated that, although the ECSA CHIKV genotype has been studied intensively, the ability of *Ae. albopictus* to transmit the Asian CHIKV genotype remains poorly characterized [17]. 

The interaction between vector and virus can impact the likelihood and magnitude of outbreaks. Vector/virus interactions are known to depend on various biotic (e.g., vector and viral genetics, vector and host competence, vector life-history traits) and abiotic (e.g., temperature, rainfall, humidity) factors [18,19,20]. Temperature is an important factor influencing vector ecology, vector competence and the extrinsic incubation period (EIP) of different viruses, including dengue virus (DENV) [21]. The ability of *Ae. albopictus* eggs to survive over winter conditions in temperate, southern Australian regions [22] and the threat of invasion of this mosquito necessitate an understanding of how lower temperatures may affect transmission of chikungunya, particularly at early time points in infection. A short EIP may necessitate faster deployment of vector control measures by public health authorities. We examined the potential for transmission at lower temperatures, using two different CHIKV genotypes, ECSA and Asian, and at 3- and 7-days post infection.

## 2. Results

### 2.1. Survival Rates

Mosquitoes were fed with infectious blood meals containing CHIKV belonging to either the ECSA or Asian genotypes at a final concentration of 1 × 10^7^ pfu/mL. They were then held at either 18 °C or 28 °C until 7 days post infection (dpi). At least 95% (number surviving/ total number of CHIKV-exposed; Table 1) of mosquitoes survived over the 7-day experiment. 

### 2.2. Effect of Temperature and Genotype on Infection, Dissemination and Transmission of CHIKV

Infection rates (number of CHIKV RNA-positive bodies/total bodies tested) of mosquitoes challenged with infectious blood meals were consistently 100%, at either 18 °C or 28 °C, and for both the ECSA and Asian genotypes (Table 2). Dissemination rates (number of CHIKV RNA-positive wings and legs/ total infected mosquitoes) were also consistently 100%, irrespective of virus genotype or temperature (Table 2). However, there was no detectable virus in saliva of either virus genotype at 3 dpi at 18 °C. At 7 dpi and 18 °C, transmissibility (number of CHIKV-positive saliva/total infected mosquitoes) was only demonstrated for the ECSA genotype and by 25% of mosquitoes. At 28 °C, the number of mosquitoes with virus in the saliva for the Asian CHIKV genotype approximately doubled, with an increasing extrinsic incubation period from 3 dpi (10.53%, 2/19) to 7 dpi (20%, 4/20) (Table 2). Similar numbers (20–25%) of mosquitoes exhibited transmissibility of the ECSA CHIKV genotype across both days. 

At 3 dpi, there was significantly higher transmissibility of the ECSA CHIKV genotype at 28 °C than 18 °C (*p* < 0.05 by Chi-Square; Table 2). There was no significant difference in transmissibility percentages between the two genotypes when mosquitoes were sampled at 7 dpi from the 28 °C treatment (Table 2). At 7 dpi, there was a significantly higher percentage of mosquitoes infected with the ECSA genotype capable of transmission in saliva than mosquitoes infected with the Asian genotype (*p* < 0.05) when held at 18 °C. At 18 °C, the ECSA CHIKV genotype displayed significantly higher transmissibility at 7 dpi than 3 dpi (*p* < 0.05). 

### 2.3. Effect of temperature and genotype on CHIKV RNA levels 

**Figure 1:**
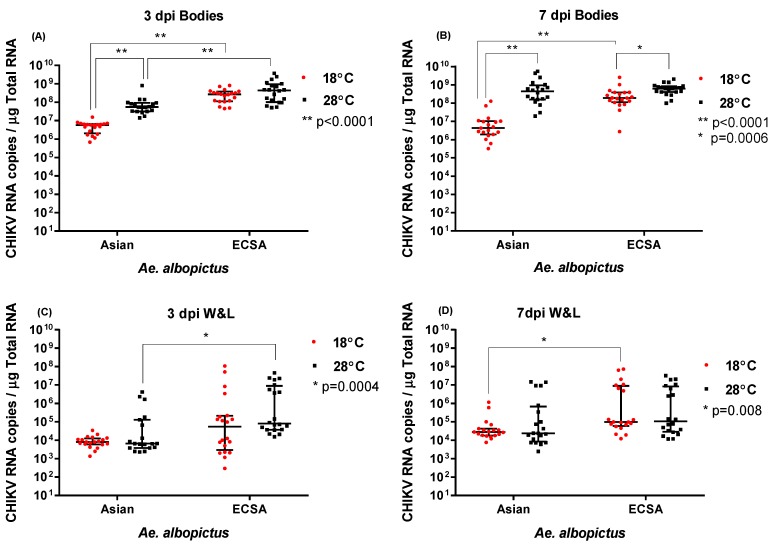
Asian and East/Central/South African (ECSA) genotype CHIKV RNA copies detected in bodies, wings and legs (**A**) CHIKV RNA copies in bodies at 18 °C and 28 °C, 3 dpi; (**B**) CHIKV RNA copies in bodies at 7 dpi across temperatures; (**C**) CHIKV RNA copies in wings (W) & legs (L) at 3 dpi across temperatures; (**D**) CHIKV RNA copies in CHIKV wings (W) & legs (L) at 7 dpi across temperatures; *denotes statistical significance (*p* < 0.05).

At 3 dpi, CHIKV load (RNA copies) in bodies of mosquitoes held at either temperature were determined by qRT-PCR. There were significantly higher viral RNA loads (1–2 logs difference) for the ECSA genotype than the Asian genotype virus at both 18 °C and 28 °C (*p* < 0.0001 by Mann-Whitney U test) (Figure 1A, Table 3). Further, there was a significantly higher viral load for the Asian genotype CHIKV at 28 °C than 18 °C (*p* < 0.0001) at 3 dpi. However, there was no statistically significant difference amongst ECSA viral loads between mosquitoes held for 3 dpi at either 18 °C or 28 °C.

Similar to the pattern observed at 3 dpi, ECSA CHIKV genotype had a significantly higher body viral load than CHIKV of Asian genotype at 7 dpi regardless of the temperature (*p* = 0.0007, Mann Whitney test). At 7 dpi and for both genotypes, significantly higher viral loads were observed in mosquito bodies at 28 °C than at 18 °C (*p* < 0.005) (Figure 1B, Table 3). Additionally, at 18 °C, CHIKV of ECSA genotype had a significantly higher body viral load than Asian CHIKV genotype (*p* < 0.0001) but, unlike the viral loads at 3 dpi, this was not the case at 28 °C. 

For wings and legs, at 3 dpi we observed significantly higher viral loads for ECSA versus the Asian genotype at 28 °C (*p* = 0.0004), but not at 18 °C (Figure 1 C). At 7 dpi, there was no significant difference in viral loads at 28 °C for either genotype. However, we observed a significant difference between genotypes at 18 °C in terms of viral loads, with ECSA being higher (*p* = 0.0008) (Figure 1 D). Notably, within each genotype, there was no significant difference in viral loads between temperatures, at either 3 dpi or 7 dpi. Overall, irrespective of the temperature, at both 3 dpi and 7 dpi, ECSA CHIKV genotype had higher viral loads in wings and legs than the Asian genotype (*p* < 0.0001 and *p* = 0.0005 respectively, Mann Whitney test). 

## 3. Discussion

We found that, irrespective of being held at either 18 °C or 28 °C, *Ae. albopictus* from Australian Torres Strait Island populations are readily susceptible to infection and dissemination with the ECSA and Asian CHIKV genotype strains used in this study. Our finding is similar to results obtained from European and American *Ae. albopictus* populations [23]. We show that Torres Strait *Ae. albopictus* had 100% infection and dissemination rates at the early time points of 3 and 7 dpi for both Asian and ECSA genotypes, at two different temperatures. A previous vector competence study conducted using mosquitoes from the Torres Strait found similar infection and dissemination rates (92%) at 28 °C for the ECSA genotype [15].

Maximum transmissibility of both Asian and ECSA genotypes was observed when mosquitoes were held at 28 °C post infection. The mosquitoes used in our study exhibited a heterogeneous pattern in the transmissibility of virus at different temperatures and time points. This heterogeneous nature of transmissibility was also observed in Brazilian *Ae. albopictus* challenged with Asian CHIKV and sampled up to 12 dpi [24]. In our study, at 28 °C, mosquitoes infected with the Asian genotype were better able to transmit virus at 7 dpi than 3 dpi. The transmissibility rates observed in our study (20–25%) are comparable to those observed in other populations, including Italian *Ae. albopictus* with percentage transmission rates of 23–36% for ECSA CHIKV [25]. A previous study of the Australian (Torres Strait) mosquito population (including *Ae. albopictus*) reported a rate of 32% when mosquitoes were held at 28 °C for 14 days post infection [15], similar to the rates we report here. 

Our results indicate that low ambient temperature delays saliva infection by CHIKV at early time points in these mosquitoes, despite high infection and dissemination rates at 3 dpi. The results suggest that both ECSA and Asian CHIKV genotypes are unable to cross the salivary gland barrier at 18 °C during early time points post infection. However, the ECSA CHIKV strain was able to evade the salivary gland barrier at low temperatures by 7 dpi, possibly mediated by the higher levels of virus observed in mosquito bodies for this genotype when compared with the Asian strain. Previous studies have also found strong interactions between virus strain and temperature on transmission efficiency of CHIKV [26].

Overall, ECSA CHIKV genotype was able to replicate to a higher viral load than the Asian genotype, consistent with ECSA CHIKV being highly adapted to *Ae. albopictus* [10]. Further, across time points and temperatures, there was an overall pattern of greater transmissibility of ECSA versus Asian CHIKV genotypes. Most surveyed populations of *Ae. albopictus* are better able to transmit the ECSA CHIKV genotype than the Asian genotype [25,27,28,29], again consistent with virus adaptation to this vector. However, Australian *Ae. albopictus* also clearly has the potential to transmit Asian CHIKV at higher temperatures, similar to *Ae. albopictus* populations from America [23]. In our study, transmissibility rates at later time points and a higher temperature of 28 °C were the same between genotypes, suggesting Asian genotype strains pose a similar risk to ECSA ones in more tropical regions of Australia. At lower temperatures encountered in more temperate areas however, our results suggest that, of the strains tested, the ECSA genotype may pose the greater risk at earlier time points, with transmission of the Asian genotype likely more delayed.

## 4. Materials and Methods 

### 4.1. Mosquitoes

*Ae. albopictus* eggs were obtained from a colony established from eggs collected on Hammond Island, Torres Strait, Australia, in July 2014 and subsequently maintained in the QIMR Berghofer insectary. Eggs were hatched and larvae reared at a density of 400 individuals in 3 L of rainwater. Larvae were provided ground TetraMin Tropical Flakes fish food (Tetra, Melle, Germany) ad libitum. Pupae were transferred to a container of rainwater inside a 30 × 30 × 30 cm cage (BugDorm, MegaView Science Education Services Co., Taichung, Taiwan) for adult emergence. Adult mosquitoes were provided with 10% sucrose solution on cotton wool pledgets and kept at 27 °C.

### 4.2. Virus Strains

CHIKV strains of the ECSA and Asian genotypes were isolated from patients returning to Australia from Mauritius in 2006 and the Caribbean in 2014, respectively. Specifically, the CHIKV strains from the ECSA and Asian genotypes used in the study are available on GenBank (accession numbers EU404186 and MF773560, respectively). Viruses were propagated in C6/36 *Ae. albopictus* cells and maintained in RPMI-1640 medium (Sigma^®^ Life Sciences, USA) supplemented with 2.5% fetal bovine serum following incubation at 28 °C for 2 days. 

Plaque assays were performed to quantify the virus titer of stock virus. Vero (African Green Monkey Kidney) cells maintained in Dulbecco’s Modified Eagle’s Medium (Sigma^®^ Life Sciences, USA) supplemented with 10% fetal bovine serum and 1% GlutaMAX™-1 (Gibco^®^ Life Technologies, USA) at 37 °C with 5% CO2 were grown until 95% confluent in the 24-well tissue culture plates. Cell monolayers were infected with a tenfold serial dilution of the sample for 2 h at 37 °C. The overlay media, consisting of a 1:1 mix of double strength Medium 199 (Gibco^®^ Life Technologies, USA) and 2% low viscosity sodium Carboxy Methyl Cellulose (Sigma^®^ Life Sciences, USA), was then added to each well. Plates were incubated for 2–3 days at 37 °C with 5% CO2 until plaques became visible. Plaques were counted following fixation and staining for 3 hours in a 0.05% crystal violet (Sigma^®^ Aldrich, USA) solution containing 1% formaldehyde (Sigma-Aldrich, USA).

### 4.3. Mosquito Oral Challenge

*Ae. albopictus* mosquitoes were challenged with CHIKV using an infectious blood meal after depriving of 10% sucrose for 36 h, and of water for 12 h prior to blood feeding. Briefly, 8 cups each containing 75–80 *Ae. albopictus* female mosquitoes, 4–7 days old, were exposed to blood meals consisting of defibrinated sheep blood (Serum Australis) and either CHIKV genotype at a final concentration of 1 × 10^7^ pfu/mL for 90 min using an artificial membrane feeding system, fitted with a porcine intestinal membrane. Exposed mosquitoes were sorted on ice and fully engorged females were maintained in a 740 FLED environmental growth chamber (HiPoint, Taiwan) set to produce a constant temperature of 18 °C or 28 °C, 70% RH and a variable light cycle (12:12 light: dark with 30 min dawn/dusk periods). The infectious blood meals were titrated as 10-fold serial dilutions on 24-well cell culture plates seeded with confluent Vero cell monolayers as described above. 

At 3 and 7 days post infection, salivation assays were performed. Briefly, 20 mosquitoes were removed at each time point, anaesthetized with CO_2_, and dissected on ice. The wings and legs of each mosquito were removed, and the saliva collected by placing the proboscis into a 200 μL pipette tip containing 40 μL of medium consisting of 10% FBS and 10% sucrose solution for 20 min. Following collection of saliva, the bodies were retained. Tubes containing the mosquito bodies, the saliva-collection media and wings and legs were stored −80 °C until assayed for virus by cell culture and/ or RNA extraction.

### 4.4. Nucleic Acid Extraction

RNA was extracted from individual mosquito bodies or wings and legs using TRIzol™ reagent (Invitrogen™, Thermo Fisher Scientific, USA). Briefly, 500 µL or 1000 µL of Trizol was added to each 2 mL screw cap vial containing the individual mosquito wings and legs or body, respectively. The samples were homogenized using a MiniBeadbeater-96 (Biospec Products, Bartlesville, Oklahoma, USA) for 90 s after adding zirconium silica glass beads (Daintree Scientific, St Helens, TAS, Australia). Total RNA was extracted from the homogenate according to the manufacturer’s specifications. RNA was dissolved in 40 µL Ultrapure™ water (Invitrogen™, Thermo Fisher Scientific, USA), and frozen at −80 °C until further analysis. 

### 4.5. Quantitative RT-PCR to Detect CHIKV

For absolute quantification of the CHIKV viral load, a control plasmid containing a cloned copy of the targeted fragment of the envelope protein E1 gene of CHIKV (nucleotides 792 to 853, GenBank accession number AM258995) was constructed. Briefly, viral RNA was extracted using the QIAamp^®^ Viral RNA Mini Kit (Qiagen, Germany) and cDNA synthesized using the SuperScript™ III Reverse Transcriptase kit (Invitrogen™, Thermo Fisher Scientific, USA) according to the manufacturer’s protocol. The targeted CHIKV fragment was amplified using CloneAmp™ HiFi PCR Premix (Takara, Clontech Laboratories, USA), and cloned into the pUC19 plasmid vector (Genscript, New Jersey, United States) using the In-Fusion^®^ Cloning Kit (Takara, Clontech Laboratories, USA) as described by the manufacturer. The presence of the insert DNA was confirmed by nucleotide sequencing. For qRT-PCR analysis, the plasmid was linearized by EcoRI (Promega, USA) and purified using the Nucleospin^®^ Gel and PCR clean-up kit (Macherey-Nagel, Germany). The concentration and purity of the linearized plasmid DNA was determined using the NanoDrop Lite spectrophotometer (Thermo Fisher Scientific, USA). The plasmid copy number was calculated based on the measured DNA concentration and its molecular weight. Plasmid DNA concentrations were confirmed prior to the preparation of a 10-fold serial dilution from 10^7^ to 10^2^ copies/µL and run in parallel with the samples in all quantitative real time PCRs (qRT-PCR). 

One-step qRT-PCR, targeting the CHIKV structural envelope glycoprotein E1 was performed to quantify CHIKV RNA present in mosquito samples. The, TaqMan^®^ Fast Virus 1-Step Master Mix (Applied Biosystems, USA) was used according to the manufacturer’s protocol. A final reaction volume of 20 μL included 2 μL RNA extract, 4 × TaqMan^®^ Fast Virus 1-Step Master Mix, 400 nM of each primer (CHIKV forward primer 5′-CCCGGTAAGAGCGGTGAA-3′, CHIKV reverse primer 5′-CTTCCGGTATGTCGATGGAGAT-3′), 250 nM of probe (5′ FAM-TGCGCCGTAGGGAACATGCC-BHQ1 3′) and Ultrapure™ water (Invitrogen™, Thermo Fisher Scientific, USA). The probe and primer pairs (Macrogen, Korea) were selected as previously described [15]. Amplification was performed in the Rotor-Gene™ Q Real-Time PCR system (Qiagen^®^, Hilden, Germany), using the thermal cycle: 50 °C for 5 min, 95 °C for 20 s, and 40 repetitions of 95 °C for 3 s and 60 °C for 30 s. A 10-fold serial dilution of linearized control plasmid DNA and negative controls (without template), were included in each run. Data were analyzed and quantified using the Rotor-Gene™ Q software (Qiagen^®^, Hilden, Germany). Samples in which CHIKV failed to amplify were classified as negative following confirmation of the presence of mosquito nucleic acid by amplification of the *Ae. albopictus* housekeeping gene RpS7 (Genbank accession number XM_019671546) using the GoTaq^®^ 1-Step RT-qPCR System (Promega, Madison, WI, USA), as per manufacturer’s recommendations, in the same Rotor-Gene PCR system. The reactions were carried out in a final reaction volume of 20 μL containing 2 µL of extracted RNA, 250 × GoScript™ RT Mix, 2 × GoTaq^®^ qPCR Master Mix, 200 nM of each RpS7 F: 5′-CTCTGACCGCTGTGTACGAT-3′, R: 5′-CAATGGTGGTCTGCTGGTTC-3′ and Ultrapure™ water (Invitrogen™, Life Technologies, USA). The cycling conditions consisted of 1 cycle at 50 °C for 5 min and 95 °C for 2 min, followed by 40 amplification cycles of 95 °C for 15 s, 60 °C for 30 s and 72 °C for 20 s. Melt curve analysis was performed to analyze the specificity of the reaction.

### 4.6. Cytopathic Effect in Cell Culture

The presence of infectious virus in mosquito saliva samples were detected as follows. In a 96-well microtiter plate, C6/36 cells were grown up to a 90% confluency in RPMI 1640 media supplemented with L-glutamine, 2.5% heat denatured FBS, 1 × Antibiotic-Antimycotic (Gibco^®^ Life Technologies, USA). Twenty µL of the saliva expectorate from each sample was inoculated into these wells and maintained at 28 °C for 5 days. The supernatants of the wells were transferred into a 24-well plate seeded with ~90% confluent monolayers of Vero cells and numbered with corresponding sample identification. Cytopathic effect was assessed after incubating the plates at 37 °C, 5% CO_2_ for 3 days. Supernatants of cells showing CPE were harvested and stored at −80 °C until confirmation of the presence of CHIKV in saliva expectorates. Briefly, viral RNA was extracted from cell culture supernatants using the High Pure Viral RNA kit (Roche diagnostics, NSW, Australia) according to the manufacturer’s specifications. qRT-PCR for the detection of CHIKV RNA were performed using the GoTaq^®^ 1-Step RT-qPCR System (Promega, Madison, WI, USA) and CHIKV-specific primers as described above. 

### 4.7. Data Analysis

Survival, infection, dissemination and transmission rates were calculated for each dpi, and for each genotype at both 18 °C and 28 °C using the equations mentioned within the results section. The statistical significance of viral loads was tested by Fisher’s exact test, Mann Whitney test along with the column statistics including median and interquartile range (IQR) and Chi square tests using GraphPad Prism^®^ Version 7.00 (GraphPad Software, La Jolla, California USA, 2008). 

## 5. Conclusions

Australian *Ae. albopictus* displayed similar infection and dissemination rates of CHIKV belonging to the ECSA and Asian genotypes, irrespective of day post infection or temperature. In our experiments, transmission at a low temperature of 18 °C was notably absent by 7 dpi for the Asian but not the ECSA CHIKV genotype. At the higher temperature of 28 °C, mosquitoes were able to transmit both genotypes equally well by day 7 dpi, compared to 3 dpi, where there was evidence of increased transmission of the ECSA genotype. In the face of the continued global spread of CHIKV and increased risk of incursion into non-endemic regions [11,12,30], our results further the understanding of the potential susceptibility of *Ae. albopictus* to different CHIKV genotypes under varied temperature conditions. Coupled with the expansion of *Aedes* mosquitoes globally, and the specific risk of invasion of *Ae. albopictus* onto mainland Australia, these findings may aid in the formulation of more effective public health management strategies for this important arbovirus. 

## Figures and Tables

**Table 1:** Proportions of surviving *Ae. albopictus* mosquitoes that were orally challenged with either Asian or ECSA genotype CHIKV, and maintained at either 18 °C or 28 °C.

dpi	Temperature	% Survival ^a^	
	°C	Asian	ECSA
3	18	100 (20/20)	100 (20/20)
	28	95 (19/20)	100 (20/20)
7	18	95 (19/20)	100 (20/20)
	28	100(20/20)	100 (20/20)

dpi = days post infection; ^a^ Percentage of surviving mosquitoes (number of surviving mosquitoes/total number of mosquitoes exposed).

**Table 2:** Infection, dissemination and transmissibility percentages of CHIKV from Asian and ECSA genotypes in *Ae. albopictus* maintained at either 18 °C or 28 °C.

dpi	Temperature	% Infection ^a^		% Dissemination ^b^		% Transmissibility ^c^	
	°C	Asian	ECSA	Asian	ECSA	Asian	ECSA
3	18	100 (20/20)	100 (20/20)	100 (20/20)	100 (20/20)	0 (0/20)	0 (0/20)
	28	100 (19/19)	100 (20/20)	100 (19/19)	100 (20/20)	10.53 (2/19)	25 (5/20) ^1^
7	18	100 (19/19)	100 (20/20)	100 (19/19)	100 (20/20)	0 (0/19)	25 (5/20) ^2,3^
	28	100 (20/20)	100 (20/20)	100 (20/20)	100 (20/20)	20 (4/20)	20 (4/20)

dpi = days post infection; ^a^ Percentage mosquitoes containing virus in their bodies (number of positive bodies/total tested); ^b^ Percentage of mosquitoes disseminating CHIKV, containing virus in their wings and legs (number of positive leg/wing samples/ total infected mosquitoes); ^c^ Percentage of mosquitoes containing virus in their saliva (number of positive saliva samples/total infected mosquitoes) and thus potentially capable of transmission; ^1^ *p* < 0.05 by Chi-Square (3 dpi 18 °C vs 28 °C ECSA); ^2^ *p* < 0.05 by Chi-Square (7 dpi 18 °C ECSA vs Asian); ^3^ *p* < 0.05 by Chi-Square (3 dpi vs 7 dpi 18 °C ECSA).

**Table 3:** CHIKV RNA copies detected in *Ae. albopictus* mosquitoes at 3 and 7 dpi.

	dpi	Genotype	18 °C		28 °C	
			Median	IQR	Median	IQR
Bodies	3	Asian	5.76 × 10^6^	2.07 × 10^6^–6.49 × 10^6^	5.46 × 10^7^	3.14 × 10^7^–9.25 × 10^7^
		ECSA	2.67 × 10^8^	1.12 × 10^8^–3.78 × 10^8^	4.39 × 10^8^	1.03 × 10^8^–9.52 × 10^8^
	7	Asian	4.31 × 10^6^	1.93 × 10^6^–1.03 × 10^7^	4.41 × 10^8^	1.60 × 10^8^–9.60 × 10^8^
		ECSA	1.91 × 10^8^	1.13 × 10^8^–3.87 × 10^8^	6.20 × 10^8^	4.07 × 10^8^–8.42 × 10^8^
Wings and Legs	3	Asian	7.88 × 10^3^	5.88 × 10^3^–1.25 × 10^4^	6.55 × 10^3^	3.86 × 10^3^–1.28 × 10^5^
		ECSA	5.44 × 10^4^	2.94 × 10^3^–2.11 × 10^5^	8.08 × 10^4^	3.70 × 10^4^–8.91 × 10^6^
	7	Asian	2.81 × 10^4^	1.83 × 10^4^–4.25 × 10^4^	2.38 × 10^4^	8.52 × 10^3^–6.65 × 10^5^
		ECSA	9.74 × 10^4^	5.98 × 10^4^–8.99 × 10^6^	1.05 × 10^5^	2.84 × 10^4^–8.28 × 10^6^

dpi = days post infection, IQR = Interquartile range.
